# Evaluating the effects of behavior change training on the knowledge, confidence and skills of sport and exercise science students

**DOI:** 10.1186/s13102-020-00209-5

**Published:** 2020-10-06

**Authors:** James Matthews, Amanda M. Hall, Alison Keogh

**Affiliations:** 1 School of Public Health, Physiotherapy, and Sports Science, University College Dublin, Dublin, Ireland; 2 Institute of Sport and Health, University College Dublin, Dublin, Ireland; 3 Faculty of Medicine, Memorial University, Newfoundland, Canada; 4 Insight Centre for Data Analytics, University College Dublin, Dublin, Ireland

**Keywords:** Behavior change, Education, Students, Communication, Training

## Abstract

**Background:**

Behavior change interventions have the potential to have a transformative effect on the health of populations. Allied health professionals have a key role to play in delivering these interventions. However, traditionally undergraduate allied health professional programs have not had a behavior change focus. The aim of this study was to assess the effectiveness of a training program on sport and exercise science students’ knowledge, confidence and skills in the provision of behavior change support.

**Method:**

A mixed method convergent design was used to address the research question. Fifteen sport and exercise science students took part in a training program consisting of seven 90-min weekly face to face group sessions. Student satisfaction with training methods was assessed. Pre-to-post training changes in students’ confidence and knowledge in the provision of behavior change support was evaluated. Delivery of behavior change support was assessed by an audio recorded role-play rated by an expert using the Health Care Climate Questionnaire, and an adapted version of the Communication Evaluation in Rehabilitation Tool. Students also completed a reflective assignment.

**Results:**

Students were satisfied with the training. There were increases in students’ confidence and knowledge of certain behavior change components post-training but not behavior change techniques. Students delivered behavior change support in a broadly needs supportive manner. The reflective assignment showed that students found particular behavior change strategies difficult to implement.

**Conclusion:**

It is feasible to train undergraduate students in particular components of behavior change support. Academic institutions should embed behavior change training into the allied health professional curriculum to ensure graduates are job ready with the knowledge, confidence and skills to support health related behavior change within the wider health system.

## Background

Physical activity can play a key role in the prevention and management of chronic disease [1]. However, levels of physical activity are low with less than 26% of men, and 19% of women meeting the recommended guidelines [2]. Consequently, there is a need to explore how individuals can be supported to change their behavior. Behavior change interventions have the potential to have a transformative effect on the health of populations [3], and allied health professionals (AHPs) have a key role to play in delivering these interventions in individual or group settings [4]. However, traditionally AHPs lack the knowledge and skills to effectively design and deliver such interventions [5, 6]. Therefore, there is a need to explore how best to train AHPs to deliver evidence-based interventions to support physical activity behavior change.

In recent times, there has been an increased emphasis on ensuring that behavior change interventions are appropriately designed, implemented and evaluated [7]. In particular, if and how theory is applied to the design of behavior change interventions and ensuring that the content of an intervention (i.e., behavior change techniques) is clearly identified and implemented [8]. While a focus on the design and content of behavior change interventions is important, there is also a need to consider how these interventions are delivered to clients, specifically through the communication style of the AHP [9]. Self-determination theory (SDT) [10] posits that the communication style utilized by people in positions of authority such as an AHP can support or undermine the basic psychological needs of a person. A needs supportive communication style is empathetic, flexible and patient whereas a needs thwarting communication style is directive, guilt-inducing and urgent [11]. This needs supportive style of an AHP facilitates people to develop autonomous motivation for the targeted behavior. Recent research suggests that interventions that emphasize a need supportive communication style are associated with long term changes in physical activity behavior [12].

Given the growing recognition of the importance of behavior change interventions within national health systems to support chronic disease prevention and management [13], it is recommended that all staff involved in activities to change people’s behavior should be appropriately trained [14, 15]. However, traditionally undergraduate programs have not had a behavior change focus [16]. While there is some evidence to support the training of AHP students in counselling techniques such as motivational interviewing [4, 17, 18], no studies have explicitly focused on training students in the design and content of behavior change interventions as well as how to delivery such an intervention. This dual focus is important because future AHPs may not only be required to deliver interventions to clients but also to design and evaluate them. Understanding the relevance of theory [19], fidelity [20] and the recognition of behavior change techniques [21] are core components of developing this behavior change competency. Furthermore, with the increasing burden on the healthcare system, there is a need to look beyond traditional AHPs to provide support for behavior change. Sport and exercise science graduates may be well placed to provide this support through programs such as exercise referral schemes. Additionally, students from this cohort frequently progress to graduate education in AHP areas such as nutrition and physiotherapy. Thus, they represent a diverse group who are likely to interact with the general public in areas related to health behavior change within their future careers. However, there are no studies conducted to date with this student cohort.

In summary, there is an increasing need to train AHPs to deliver health related behavior change interventions. This training should be formally embedded within the curriculum of undergraduate programs to ensure students are competent to support clients after graduation. However, few studies have assessed the feasibility and effectiveness of behavior change training with this cohort of students. Therefore, the aim of this study was to assess the impact of a training program on sport and exercise science students’ knowledge, confidence and skills in behavior change.

## Method

### Study design and research ethics

This was a single arm, non-randomized pre-post study which took place within the context of a final year undergraduate degree program at a large university in Ireland. A mixed method convergent design was used in this study as it was believed that the integration of both quantitative and qualitative data would enable a more thorough answer of the research question [22]. Specifically, quantitative and qualitative data were analysed separately, and then merged at the results stage to see if data converged or not regarding the students’ use of the SDT-based communication style [23]. Ethical approval was granted from the University’s Human Research Ethics Committee (LS-E-18-182).

### Participants and procedure

Participants were final year sport and exercise science students. The students that chose to participate provided written consent. A research assistant collected and anonymized all participant data prior to data analysis.

### Program description

The program consisted of seven 90-min weekly face to face sessions delivered over a ten-week period and was broken into four distinct yet interrelated sections that targeted the design, delivery and evaluation of behavior change interventions (Table 1). The program was delivered by two researchers, a chartered Psychologist and a chartered Physiotherapist. Both researchers had PhDs related to behavior change and had published research on behavior change and physical activity in peer-reviewed journals in the last 3 years. Section 1 focused on behavior change theory which included the behavior change wheel [24] and the theoretical domains framework (TDF) [25]. Section 2 focused on behavior change techniques, and how to identify, describe and use particular techniques based on the behavior change technique taxonomy v1 [8]. Section 3 introduced how to use a client centred SDT-based communication style to deliver behavior change to individuals and groups (see the supplemental files for a description of the particular strategies). Section 4 examined the importance of intervention fidelity and how to assess fidelity within behavior change interventions. The program was developed from a social constructivist viewpoint [26] where the researchers acted as facilitators promoting peer interaction and collaboration. To create authentic and meaningful learning experiences, the students were asked to work in groups both in and outside of sessions [27].

**Table 1:** Behavior change training program structure and content

Section	Time spent	Section learning outcomes	Sample content and materials
Behavior change theory, and mechanisms of action	3 h	1. Apply a systematic behavior change design process 2. Use the Behavior Change Wheel (BCW) to support the design process 3. Diagnose what needs to change to allow the targeted behavior to occur 4. Use the Theoretical Domains Framework (TDF) to support behavioral diagnosis	1. Information provided on behavior change interventions, and the challenges in changing people’s behavior. 2. Information provided on the important of theoretical frameworks (e.g., BCW) in designing interventions, and how to target mechanisms of action (e.g., TDF). 3. Personal reflection on students’ experience in attempting to change their own behavior. 4. Group discussions on students’ awareness of the conditions required to change behavior. 5. Case studies completed in groups to practice behavioral diagnosis for physical activity in various populations. 6. Interactive quizzes to reinforce components of the BCW and the TDF.
Behavior change techniques	3 h	1. Define a behavior change technique 2. Select appropriate behavior change techniques for an intervention 3. Identify behavior change techniques within intervention descriptions 4. Apply behavior change techniques in a real-world situation	1. Information provided on behavior change techniques and their role within behavior change interventions. 2. Information provided on the range of behavior change techniques within the Behavior Change Taxonomy V1 (Michie et al., 2013), and the evidence for their use with physical activity. 3. Personal reflection on students’ experience in using selected behavior change techniques. 4. Both individual and group activities using the online training tool (https://www.bct-taxonomy.com/) to identify behavior change techniques within interventions. 5. Group discussions on how students’ can select and use behavior change techniques within an intervention targeting physical activity.
Client-centred communication	3 h	1. Describe a client centred communication approach 2. Apply a client centre communication style in a real-world situation 3. Critically reflect on their application of SDT-based communication strategies	1. Information provided on the importance on client-centred communication within behavior change interventions. 2. Information provided on the needs supportive SDT-based communication strategies to facilitate client behavior change, and the evidence for their use with physical activity. 3. Personal reflection on students’ experience of a needs supportive or needs thwarting communication style from an authority figure. 4. Observation and comment on video interactions between an allied health professional and a client. 5. Group discussion on the barriers and enablers to using a needs supportive communication style. 6. Multiple role plays to practice the use of needs supportive communication strategies with peer and facilitator feedback.
Intervention evaluation	1.5 h	1. Define intervention fidelity 2. Recognise the five domains of fidelity 3. Apply fidelity assessment techniques within a behavior change intervention	1. Information provided on the importance on assessing fidelity within behavior change interventions. 2. Group discussion on the barriers and enablers to assessing fidelity within behavior change interventions. 3. Case studies completed in groups to practice the assessment of intervention fidelity for physical activity interventions. 4. Interactive quiz to identify different intervention fidelity strategies.
Materials	Brief PowerPoint lectures; Pre-reading materials for each session; Videos; Group activities, Case studies; Interactive quizzes (www.kahoot.it); Session handouts		

### Outcome measures

Adopting a similar approach to a recent study with physiotherapists [28], the effectiveness of the program was assessed using the Kirkpatrick model of evaluation at the levels of reaction and learning [29]. Briefly, “reaction” assesses learners’ satisfaction with the learning experience, training methods, materials and quality of instruction. “Learning” explores the modification of learners’ knowledge and confidence, and an increase in their skills. The measurement tools used are described in detail in the supplemental files and are briefly outlined below.

#### Reaction

Students’ satisfaction with the program and related components was measured with a study specific questionnaire following completion of the program. This questionnaire had nine statements which were answered on a 5-point Likert scale ranging from 1 “*strongly disagree*” to 5 “*strongly agree*”. Students’ engagement with the program was further evaluated by recording attendance at each session.

#### Learning

Learning was assessed in a number of different ways. Students’ confidence in applying these behavior change components was evaluated using a four-item study specific questionnaire, measured on a 7-point Likert scale ranging from 1 “*not at all confident*” to 7 “*very confident*” . Then, students’ knowledge of the behavior change components pre and post-training was assessed. First, students’ knowledge of SDT-based client centred communication strategies was evaluated using a narrative case study as has been used in previous studies [28]. Students were asked to briefly record how they would interact with a person who was not physical activity. Two blinded raters independently assessed students’ responses for the presence or absence of the 11 SDT-based communication strategies (see the supplemental files for a description of each strategy), with a score of “1″ for strategies that were present and “0″ for strategies that were absent. Students’ knowledge of behavior change techniques was then assessed by providing them with a short intervention description (adapted from the online behavior change technique taxonomy site; www.bct-taxonomy.com) and a list of six possible behavior change techniques that may be present in this description. Students were asked to identify if any of these behavior change techniques were present in the intervention description. Two blinded raters again independently assessed the responses, with a score of ‘2’ provided when students correctly identified a behavior change technique and − 1 if an incorrect behavior change technique was listed by students.

Students’ skills were also assessed with a simulated physical activity counselling session. However, this component of the assessment process was only completed post-training. The lack of a pre-training assessment for this component was primarily due to a lack of time and the need to complete the program before the end of the academic semester. Thus, it was not possible to give students sufficient time to conduct a physical activity counselling session prior to beginning the program. For these sessions, it was deemed not appropriate for students to work with real clients at this stage of learning, and it was also not feasible to use trained simulated clients due to cost constraints. Therefore, students were asked to apply their learning in a simulated session post-training with a family member or friend acting as a hypothetical client. The use of a family member or friend in simulated sessions has occurred in similar programmes [30]. Students audio recorded their interaction with the hypothetical client who was not currently physically active but who was considering changing their behavior. Each audio recording was coded by a blinded independent rater to assess students’ use of a client centred communication style, and specific behavior change strategies. Four (27%) of these audio recordings were double coded by a second blinded rater. Prior to coding, both raters completed a training and familiarization process to support consistent and accurate coding.

Measuring competency in communication skills can be challenging [31]. For this study, we chose the Health Care Climate Questionnaire (HCCQ) [32] as the primary measure to assess provider delivery of the client centred communication style. An adapted version of the Communication Evaluation in Rehabilitation Tool (CERT) [33] was used as a secondary measure. We selected these two scales because they have demonstrated adequate reliability and validity and have been used in similar contexts [33, 34]. The selection of the HCCQ enabled us to potentially compare students’ scores in the present study with participants’ scores in previous studies (whilst acknowledging the differences between cohorts and contexts in doing so). We also chose the mid-point of each scale as demonstrating competence. The selection of the mid-point was based on previous studies using the HCCQ where healthcare professional scores just above the mid-point have been linked to changes in client motivation [35, 36]. Furthermore, the mid-point is also used as a competency threshold in other communication related measures such as the motivational interviewing treatment integrity tool [37]. However, it must acknowledged that while the selection of the mid-point of the scale as a measure of competency is usually based on expert opinion, it is still a somewhat arbitrary selection with the need for further assessment of its validity to support its use [4]. As part of this process, students were asked to complete a reflective assignment on their experience of conducting a physical activity counselling session. Students were asked to listen back to the audio recording of the session and complete this reflective assignment within 24 h of completing the session. Specifically while listening to the audio recording, students were asked to reflect on what was good or challenging about their session, and also what they would do differently in any future consultation. Students then submitted their audio recording and reflection to a research assistant who anonymized the data prior to analysis.

### Data analysis

Excel (Microsoft for Mac, version 14.2.3) and a statistical software package (IBM, SPSS Statistics, version 24) were used to analyze the quantitative data in this study.

#### Reaction

Students’ satisfaction with the program and related components was assessed using descriptive statistics.

#### Learning

For this level of the Kirkpatrick model of evaluation, a similar analysis process to a recent study was adopted [28]. First, descriptive statistics were used to calculate scores pre and post-training for students’ confidence in using the particular behavior change components. Differences between these scores were calculated using the Wilcoxon signed-rank tests and adjusted. Then the number of SDT-based strategies used by each student in the narrative case studies and the percentage of students who used each strategy was calculated, with the differences in the use of each strategy from pre to post-training assessed using McNemar’s test. A similar approach was used for assessing students’ knowledge of behavior change techniques, with differences in behavior change technique recognition pre and post-training also determined using McNemar’s test. For both of these assessments, interrater agreement was calculated using percentage agreement, and all results were adjusted for multiplicity using Bonferroni corrections. The final component analyzed at this level was students’ use of a client centred communication style during their simulated physical activity counselling session. To do so, descriptive statistics were calculated based on students’ scores on the HCCQ and the adapted version of the CERT. For this component, 27% of the sessions were double coded, and agreement between the two blinded raters was established using an intraclass correlation coefficient 2-way random model for absolute agreement.

Qualitative data from the students’ reflections were analyzed using deductive thematic analysis [38]. This deductive approach to thematic analysis allows for coding to be top down and fitted to a pre-existing coding framework [38]. The CERT scale was used as the framework for this analysis and codes were aligned to the framework, and then linked to two general dimensions. Dimensions, themes and illustrative quotes were then presented to a member of the research team who acted as a critical friend, discussing their interpretation with the lead author and providing feedback. In line with a mixed method convergent design, following separate analysis of the quantitative and qualitative data for the simulated physical activity counselling session, the two data sets were merged to attempt to assess the impact of the program on students’ use of a SDT-based communication style in this simulated session.

## Results

A total of 15 students completed pre and post-program assessments (7 females; 46.7%). These students attended a median of 6 out of a possible 7 sessions, representing 86% of sessions.

### Reaction

Overall, students were satisfied with the program (median = 3.8 [0.6; 2.9–4.7]). While students reported being very satisfied with nearly all components of the program, they were less than satisfied with the use of the online behavior change technique training tool (median = 2.0 [1.8; 1.0–5.0]).

### Learning

Students’ overall confidence in applying behavior change components significantly increased post-training (z score = − 2.5; *p* = 0.013). Two items retained significance following Bonferroni correction, i.e., confidence to recognise and apply an appropriate theory to guide an intervention (z score = 3.0; *p* = 0.003) and confidence to select appropriate behavior change techniques to target constructs (z score = 2.9 *p* = 0.004) (see supplemental files for further details). Students’ knowledge of the SDT-based client centred strategies again increased post-training but did not retain significance when a Bonferroni correction was applied. Although improvements in the use of individual strategies were observed from pre to post-training, none demonstrated significance when Bonferroni adjustments were applied (Table 2). There was no significant change in students’ knowledge of behavior change techniques from pre to post-training. Non-significant improvements were seen in students’ recognition of the behavior change technique 12.5 (‘*adding objects to the environment*’). Students were also less likely to apply incorrect behavior change techniques to the intervention description post-training.

**Table 2:** Changes in (i) students’ knowledge of SDT-based communication strategies and (ii) behavior change techniques following completion of the program

Component	Median pre-training (IQR; min-max)	Median post-training (IQR; min-max)	Z score^**a**^	***p***-value
**Knowledge of SDT-based communication strategies total** **[possible score of 11]** ^**b**^	**3.0 (2.0; .0–3.0)**	**4.0 (2.0; .0–6.0)**	**2.5**	**0.01**
1. Open questions [number, % used in response]	4 (26.7%)	10 (66.7%)	NA^d^	0.07
2. Staying silent [% used in response]	0 (0.0%)	5 (33.3%)	NA^d^	0.06
3. Summaries [% used in response]	0 (0.0%)	4 (26.7%)	NA^d^	0.13
4. Reflection [% used in response]	0 (0.0%)	6 (40.0%)	NA^d^	0.03
5. Asking permission [% used in response]	0 (0.0%	1 (6.7%)	NA^d^	1.00
6. Providing a meaningful rationale [% used in response]	7 (46.7%)	5 (33.3%)	NA^d^	0.69
7. Opportunities for client input/choice [% used in response]	4 (26.7%)	4 (26.7%)	NA^d^	1.00
8. Autonomy supportive language [% used in response]	4 (26.7%)	4 (26.7%)	NA^d^	1.00
9. Goal setting [% used in response]	5 (33.3%)	8 (53.3%)	NA^d^	0.45
10. Barrier identification [% used in response]	3 (20.0%)	6 (40.0%)	NA^d^	0.45
11. Solution identification [% used in response]	4 (26.7%)	4 (26.7%)	NA^d^	1.00
**Knowledge of behavior change techniques (BCT) total** **[possible score of 6]**^**c**^	**3.5 (2.3; 0.0–6.0)**	**4.0 (4.3; 1.0–6.0)**	**0.8**	**0.41**
1. BCT 2.2 provide feedback on behavior [% correct]	12 (85.7%)	10 (71.4%)	NA^e^	0.63
2. BCT 5.1 information on health consequences [% correct]	9 (64.3%)	7 (50.0%)	NA^e^	0.69
3. BCT 12.5 adding objects to the environment [% correct]	9 (64.3%)	14 (100%)	NA^e^	0.06
4. Application of incorrect BCTs [% present]	9 (64.3%)	5 (35.7%)	NA^e^	0.18

^a^ Difference between pre and post-program results;

^b^ Individual questions listed as the percentage of students who answered the question correctly, and/or included a strategy in their response;

^c^ Pre and post-BCT data relates to 14/15 students due to missing data;

^d^ Nominal data therefore McNemar’s test calculated; *: *p* < 0.007 is significant following Bonferroni correction; ** *p* < 0.01 is significant following Bonferroni correction

Students engaged in the simulated physical activity counselling session in a manner consistent with a client centred communication style (HCCQ: median = 5.0 [IQR = 1.17 min – max = 1.67–6.67]; CERT: median = 4.73, [IQR = 1.36, min – max = 2.09–6.27]; Table 3). The median scores of the 11 SDT-based strategies were delivered during the counselling session at or above the set competence level (Table 3). The text from the reflective assignments broadly aligns with the independent rater’s assessment of the audio recordings (Table 4). In general, students perceived that they had used open questions and summaries well during the counselling session. However, they reported greater difficulty implementing complex reflections with their clients. Some students also reported forgetting to seek permission to give advice. While students felt they had provided opportunities for their client to contribute to the conversation, some reported struggling with the provision of autonomy support. Most students reflected on how they engaged in goal setting with their clients. However, a number of the students discussed how this process was not sufficiently detailed, leaving only a vague sense of a plan at the end of the session. When students were asked to look forward and record what they might do differently, they focused on ensuring they sought permission from the client to give advice, promoting even greater opportunities for client input through open questions, and the use of a more focused collaborative goal-setting process.

**Table 3:** Students’ delivery of a needs supportive communication style and the SDT-based communication strategies during the audio-recorded simulated physical activity counselling session

Measure	Median (IQR; min-max)
**Health Care Climate Questionnaire total [1 “not at all well’ to 7 “very well”]**	**5.0 (1.2; 1.7–6.7)**
**Modified CERT scale total [1 “not at all well’ to 7 “very well”]**	**4.7 (1.4; 2.1–6.3**)
1. Open questions [1–7]	6.0 (1.5; 3.0–7.0)
2. Staying silent [1–7]	5.0 (1.5; 3.0–7.0)
3. Summaries [1–7]	6.0 (1.5; 1.0–6.0)
4. Reflection [1–7]	4.0 (1.5; 2.0–6.0)
5. Asking permission [1–7]	5.0 (1.5; 1.0–6.0)
6. Providing a meaningful rationale [1–7]	4.0 (2.5; 1.0–6.0)
7. Opportunities for client input/choice [1–7]	5.0 (2.0; 4.0–7.0)
8. Autonomy supportive language [1–7]	5.0 (1.5; 2.0–6.0)
9. Goal setting [1–7]	4.0 (2.5; 2.0–7.0)
10. Barrier identification [1–7]	5.0 (2.0; 2.0–6.0)
11. Solution identification [1–7]	5.0 (2.0; 1.0–7.0)

**Table 4:** Students’ reflections on the counselling session

Dimension 1. Looking back...Thoughts, feelings and evaluation of the session		
**Theme**	**Strategy**	**Exemplar quote**
Engage	Open questions	“I made good use of open questions. I feel like this increased my understanding of how the client felt, along with making the client feel understood.” (Student 6)
	Staying silent	“Sometimes I interrupted or clipped the end of the client’s sentences which is a personal weakness …” (Student 11)
	Summarising	“I thought in particular that my use of summaries was effective as they added to what was already said as opposed to just repeating what had been said.” (Student 7)
	Reflection	The use of simple reflections also allowed me to yield more information (Student 15) I failed to use any reflections in order to dig a bit deeper into her lack of physical activity which may have yielded more insight (Student 14)
	Asking permission	“However, I failed to ask permission, when focusing on physical activity rather than diet, and in general failed to ask the client’s opinion on the direction of the conversation.” (Student 9)
Guide	Provide rationale	“I feel I appropriately requested permission before extending rationale for the change. However, I should have been less vague and provided more comprehensive reasoning.” (Student 8)
	Opportunities for input	“I thought I explored her options with her in a manner that supported her autonomy in so far as she was often the one coming up with the suggestions herself with me as the practitioner providing guidance more than anything.” (Student 7
	Autonomy supportive behavior	“I feel suggestions were flexible and offered alternatives. Time and space to explore and have autonomy over their choices was given.” (Student 4) “Instead of letting him decide if he wanted to set a goal I asserted we will set a goal.” (Student 15)
Plan	Goal-setting	“I feel like our discussion was quite vague and I’m questioning if we should have come up with a more concrete plan. I’m unsure as to how well I strengthened my client’s commitment to the plan and feel this is a skill I need.” (Student 6) “I feel I could have done better regarding planning. Although I discussed the “when” of the client’s short-term goal of looking into exercise classes, I failed to address the specificity and objectivity of the overall goal of increasing physical activity, meaning the client still lacked a clear plan.” (Student 9)
	Identify barriers	Potential barriers were not mentioned and client understanding was not present (Student 12)
	Problem-solving	“But the client should have had more involvement. I think I was too quick to suggest solutions and the client would have benefited from an open question leading them to investigate ways around the barriers.” (Student 8)
**Dimension 2. Looking forward...**w**hat would they do differently?**		
**Theme**	**Strategies**	**Exemplar quote**
Engage	Open questions	“Next time I would use more open questions as opposed to closed ones especially towards the beginning of the conversation, allowing the client to lead the conversation.” (Student 14)
	Summarising	“In future, I need to summarise what the client is saying more regularly during the interview to ensure we are on the same wavelength and avoid any miscommunication about the situation.” (Student 5)
	Reflection	“Open questions and summaries were used effectively but I think that in the future I should use reflections more.” (Student 7)
	Asking permission	“I need to remember to ask permission before offering advice, so that the client sees it as a suggestion rather than a decision that is forced upon them.” (Student 4) “There were a number of occasions where I gave advice and didn’t ask permission which is something that should be improved for the future.” (Student 13)
Guide	Opportunities for input	“however in future I should allow the client to provide more input.” (Student 2) “In the future I think it would be best not just to ask the client if they were ok with what I suggested, but also if they had anything to add.” (Student 6)
	Autonomy supportive behavior	“I think that in the future, it is important to try and evoke the client’s own motivation for change rather than me trying to offer a reason for them to do it.” (Student 5)
Plan	Goal-setting	“In future, I think the client needs to be more involved in the goal setting process, I was telling him, rather than him suggesting how to do it. If he chooses a specific goal it will lead to increased commitment.” (Student, 7)

## Discussion

The aim of this study was to assess the impact of a training program on sport and exercise science undergraduate students’ knowledge, confidence and skills in the provision of behavior change support. Overall, students were satisfied with the program. Results indicated that students’ confidence increased from pre to post-training. Students’ skills were influenced positively by the training program as they delivered the simulated physical activity counselling session using a client centred communication style to a competent level. Students’ self-assessment of their performance in the simulated counselling session broadly aligned with the independent raters’ assessment. This study has demonstrated that while it is possible to train undergraduate students in particular components of behavior change, refinements are required to enhance this training process further.

In general, students were positive about the training program. However, they were dissatisfied with the use of the online behavior change technique training tool (www.bct-taxonomy.com). Anecdotally, students reported some issues with functionality, and how the tool lacked direct relevance as to how they might use behavior change techniques within their future practice. More encouragingly, students did report intending to use the behavior change components in their future professional careers. Alongside satisfaction with the training program, students’ confidence in using the particular behavior change components increased post-training. This is important as it can provide an indication as to how likely a learner might be to utilize the knowledge and skills they have developed in the future [29]. However, there were limited changes in students’ knowledge of the SDT-based strategies or behavior change techniques. This lack of change in students’ knowledge of SDT-based strategies mirrors the findings of a previous research study [28]. For behavior change techniques, this lack of change may have been due to the type of learning and evaluation methods applied in this section of the training program. The training followed the online tool (www.bct-taxonomy.com) where a focus is placed upon the recognition of behavior change techniques in intervention descriptions. This process is highly structured with the purpose being to teach individuals to recognise a behavior change technique as it is defined in the behavior change technique taxonomy [39]. However as discussed above, students disliked the use of this online training tool and this dissatisfaction may have influenced their performance on this particular post-test assessment. Indeed, our findings somewhat mirror recent research which suggests that this form of behavior change technique training may not always improve individuals’ abilities to recognise behavior change techniques [40]. Therefore, there is a need to reconsider how behavior change techniques are taught to students in a manner that not only improves their knowledge but also their competency in applying behavior change techniques in a practical setting.

With respect to the enhancement of skills, students were judged to have delivered the simulated physical activity counselling session in a client centred manner. Indeed, students’ scores on certain measures (i.e., HCCQ) were higher than reported in some previous studies with experienced healthcare professionals [35]. For example, for the HCCQ, the median score in this study was 5 (out of 7) whereas the mean score in Murray et al. [35] with physiotherapists was 4.67 (out of 7). This discrepancy in performance is likely explained by the simulated nature of the counselling session in the present study. Specifically, students employed family members and friends to act as clients as compared to the healthcare professionals who would have engaged with actual clients in these other studies. As noted in previous research [30], it is possible that family members and friends provided an “easier” simulated session, and that their stories may have been quite artificial. Nonetheless, this simulated session has initiated students’ development of SDT-based strategies in a safe environment and enabled them to demonstrate their relative competence. Future studies could consider a scaffolded approach to the practice of these skills from in-class to simulated clients outside of the classroom setting, to actual clients to mirror the reality of delivering behavior change in a real-world setting. Looking at the particular strategies, students seemed to struggle more with certain strategies as compared to others, for example, reflections and collaborative goal-setting. The provision of reflections in which the practitioner is meant to convey understanding and in the case of complex reflections add substantial meaning to what the client has said is challenging. Our results are in line with recent research in which students struggled to reach proficiency for complex reflections [4]. Interestingly, researchers have suggested that the use of simple reflections may be as effective as complex reflections to encourage client discussion of changing behavior [41]. Furthermore, students found goal-setting somewhat difficult to implement with clients, a finding that is mirrored in real-world clinical settings [42]. Consequently, there needs to be further training on how to implement collaborative goal-setting beginning at undergraduate level alongside practical tools such as “goal cards” which may aid the process [43].

The self-reflection component of the training program appears to have been beneficial with students reporting it a useful component of the process. Students’ reflections also broadly aligned with the coding by the independent rater and provided further context to the findings which could inform future training programs. Self-reflection promotes active engagement by students in developing their skills in planning, monitoring and evaluating their learning [44]. These skills are not only important as students but as future AHPs who will need to be able to monitor and effectively update their knowledge and skills for the new and demanding tasks which they may face in fast changing and resource challenged health systems. Looking forward, it may be useful to broaden out the assessment process to include peer assessment. This would further support students’ critical thinking skills by forming evaluative judgements of others’ work, and the generation of information rich feedback to their peers based on the evidence [44].

### Limitations and future research

There were several limitations to the study. The use of family members and friends in the simulated counselling sessions rather than real clients or actors while an accepted practice [45] was a limitation and may have led to the higher ratings on measures such as the HCCQ as compared to other studies. Thus, the use of real or trained simulated clients in future studies should be considered. No pre-training audio recording assessment was completed. Therefore, it was not possible to assess whether students’ communication skills improved over time. Future studies should include the use of a pre-post design to ensure any change in communications skills over time can be assessed. Finally, future research evaluating behavior change training should include an equivalent control group.

## Conclusion

The study findings highlight how sport and exercise science students can be trained in aspects of behavior change support within an undergraduate curriculum. To ensure that students are proficient, there is a need to embed behavior change training throughout the curriculum and not just within a single semester. Indeed, it should be scaffolded through the curriculum with students developing knowledge and skills in the formative stages of learning before being given the opportunity to apply these skills in “real world” settings through clinical or community-based placements.

AHPs have a key role to play in supporting individuals to initiate and maintain changes to their behavior such as physical activity which can reduce chronic disease incidence. Academic institutions have an important role to play in ensuring that their graduates are job ready with the knowledge, confidence and skills to support health related behavior change within the wider health system**.**

## Supplementary information

**Additional file 1.**

## Data Availability

The dataset used in the current study is available from the corresponding author on request.

## Abbreviations

AHPs: Allied health professionals
TDF: Theoretical domains framework
BCW: Behavior Change Wheel
HCCQ: Health Care Climate Questionnaire
CERT: Communication Evaluation in Rehabilitation Tool

## References

[1] Warburton D, Whitney CN, Bredin S (2006). Health benefits of physical activity: the evidence. Can Med Assoc J.

[2] Piercy KL, Troiano RP, Ballard RM, Carlson SA, Fulton JE, Galuska DA, Olson RD (2018). The physical activity guidelines for Americans physical activity guidelines for Americans physical activity guidelines for Americans. J Amer Med Assoc.

[3] Cecchini M, Sassi F, Lauer JA, Lee YY, Guajardo-Barron V, Chisholm D (2010). Tackling of unhealthy diets, physical inactivity, and obesity: health effects and cost-effectiveness. Lancet.

[4] Fortune J, Breckon J, Norris M, Eva G, Frater T. Motivational interviewing training for physiotherapy and occupational therapy students: effect on confidence, knowledge and skills. Patient Educ Couns. 2018. 10.1016/j.pec.2018.11.014. 10.1016/j.pec.2018.11.014 30482468

[5] Freene N, Cools S, Bissett B (2017). Are we missing opportunities? Physiotherapy and physical activity promotion: a cross-sectional survey. BMC Sports Sci Med Reh.

[6] Geidl W, Wais J, Fangmann C (2019). Physical activity promotion in daily exercise therapy: the perspectives of exercise therapists in German rehabilitation settings. BMC Sports Sci Med Rehabil.

[7] Michie S, Carey RN, Johnston M, Rothman AJ, de Bruin M, Kelly MP, Connell LE (2018). From theory-inspired to theory-based interventions: a protocol for developing and testing a methodology for linking behavior change techniques to theoretical mechanisms of action. Ann Behav Med.

[8] Michie S, Richardson M, Johnston M, Abraham C, Francis J, Hardeman W (2013). The behavior change technique taxonomy (v1) of 93 hierarchically clustered techniques: building an international consensus for the reporting of behavior change interventions. Ann Behav Med.

[9] Hardcastle SJ. Commentary: Interpersonal style should be included in taxonomies of behavior change techniques. Front Psychol. 2016;7(894). 10.3389/fpsyg.2016.00894. 10.3389/fpsyg.2016.00894 PMC4909760 27378325

[10] Deci E, Ryan R (2000). The ‘what’ and ‘why’ of goal pursuits: human needs and the self-determination of behavior. Psychol Inq.

[11] Ntoumanis N, Quested E, Reeve J (2017). Need supportive communication: implications for motivation in sport, exercise and physical activity. Persuasion and communication in sport, exercise and physical activity.

[12] Samdal GB, Eide GE, Barth T, Williams G, Meland E (2017). Effective behavior change techniques for physical activity and healthy eating in overweight and obese adults; systematic review and meta-regression analyses. Int J Behav Nutr Phys Act.

[13] Chronic Conditions Working Group of the Health Service Executive (2017). Living well with a chronic condition.

[14] Sinclair D, Savage E, O’ Brien M, O’Reilly A, Mullaney C, et al. Developing a national undergraduate standardized curriculum for future healthcare professionals on “Making Every Contact Count” for chronic disease prevention in the Republic of Ireland. J Interprof Care. 2019. 10.1080/13561820.2019.1684884. 10.1080/13561820.2019.1684884 31762372

[15] NICE (2014). Behavior change: Individual approaches (PH49).

[16] O’May F, Gill J, McWhirter E, Kantartzis S, Rees C, Murray K (2016). A teachable moment for the teachable moment? A prospective study to evaluate delivery of a workshop designed to increase knowledge and skills in relation to alcohol brief interventions (ABIs) amongst final year nursing and occupational therapy undergraduates. Nurse Educ Pract.

[17] Norris M, Eva G, Fortune J, Frater T, Breckon J (2019). Educating undergraduate occupational therapy and physiotherapy students in motivational interviewing: the student perspective. BMC Med Educ.

[18] Simper TN, Breckon J, Kilner K (2019). Effectiveness of training final-year undergraduate nutritionists in motivational interviewing. Patient Educ Couns.

[19] Prestwich A, Sniehotta FF, Whittington C, Dombrowski SU, Rogers L, Michie S (2014). Does theory influence the effectiveness of health behavior interventions? Meta-analysis. Health Psychol.

[20] Borelli B (2011). The assessment, monitoring and enhancement of treatment fidelity in public health clinical trials. J Public Health Dent.

[21] Johnston M (2014). Improving the reporting of behavior change interventions. Eur Health Psychol.

[22] Barbour RS (1999). The case for combining qualitative and quantitative approaches in health services research. J Health Serv Res Policy.

[23] Creswell JW, Plano Clark VL (2011). Designing and conducting mixed methods research.

[24] Michie S, van Stralen MM, West R (2011). The behavior change wheel: a new method for characterising and designing behavior change interventions. Implement Sci.

[25] Cane J, O’Connor D, Michie S (2012). Validation of the theoretical domains framework for use in behavior change and implementation research. Implement Sci.

[26] Sun H, Chen A (2010). Pedagogical understanding of the self-determination theory in physical education. Quest..

[27] Palincsar A (1998). Social constructivist perspectives on teaching and learning. Annu Rev Psychol.

[28] Keogh A, Matthews J, Segurado R, Hurley DA (2018). Feasibility of training physical therapists to deliver the theory-based self-Management of Osteoarthritis and low Back Pain through Activity and skills (SOLAS) intervention within a trial. Phys Ther.

[29] Kirkpatrick D, Kirkpatrick J (2006). Evaluating training programs: the four levels.

[30] Schoo AM, Lawn S, Rudnik E, Litt JC (2015). Teaching health science students foundation motivational interviewing skills: use of motivational interviewing treatment integrity and self-reflection to approach transformative learning. BMC Med Educ.

[31] Spitzberg B (2013). (re) introducing communication competence to the health professions. J. Public Health Res.

[32] Williams GC, Grow VM, Freedman ZR, Ryan RM, Deci EL (1996). Motivational predictors of weight loss and weight-loss maintenance. J Pers Soc Psychol.

[33] Murray A, Hall AM, Williams GW, McDonough SM, Ntoumanis N, Taylor I, Matthews J (2019). Assessing physiotherapists’ communication skills for promoting patient autonomy for self-management: reliability and validity of the communication evaluation in rehabilitation tool. Disabil Rehabil.

[34] Williams GC, Deci EL (2001). Activating patients for smoking cessation through physician autonomy support. Med Care.

[35] Murray A, Hall A, Williams G, Mcdonough S, Ntoumanis N, Taylor I, Jackson B (2015). Effect of a self-determination theory - based communication skills training program on physiotherapists' psychological support for their patients with chronic low back pain: a randomized controlled trial. Arch Phys Med Rehabil.

[36] Lonsdale C, Hall AM, Murray A, Williams GC, McDonough SM, Ntoumanis N, Owen K, Schwarzer R, Parker P, Kolt GS, Hurley DA. Communication skills training for practitioners to increase patient adherence to home-based rehabilitation for chronic low Back pain: results of a cluster randomized controlled trial. Arch Phys Med Rehabil. 2017. 10.1016/j.apmr.2017.02.025. 10.1016/j.apmr.2017.02.025 28363702

[37] TB TBM, Rowell LN, Manuel JK, Ernst D, Houck JM (2016). The motivational interviewing treatment integrity code (MITI 4): rationale, preliminary reliability and validity. J Subst Abus Treat.

[38] Braun V, Clarke V (2006). Using thematic analysis in psychology. Qual Res Psychol.

[39] Wood CE, Richardson M, Johnston M, Abraham C, Francis J, Hardeman W, Michie S (2014). Applying the behavior change technique (BCT) taxonomy v1: a study of coder training. Transl Behav Med.

[40] Wood CE, Richardson M, Johnston M, Abraham C, Francis J, Hardeman W, Michie S (2016). Reporting behavior change interventions: do the behavior change technique taxonomy v1, and training in its use, improve the quality of intervention descriptions?. Implement Sci.

[41] Apodaca TR, Jackson KM, Borsari B, Magill M, Longabaugh R, Mastroleo NR, Barnett NP (2016). Which individual therapist behaviors elicit client change talk and sustain talk in motivational interviewing?. J Subst Abus Treat.

[42] Rose A, Rosewilliam S, Soundy A (2017). Shared decision making within goal setting in rehabilitation settings: a systematic review. Patient Educ Couns.

[43] Rose G, Smith L (2018). Mental health recovery, goal setting and working alliance in an Australian community-managed organisation. Health Psychol Op.

[44] Panadero E, Broadbent J, Boud D, Lodge J (2019). Using formative assessment to influence self- and co-regulated learning: the role of evaluative judgement. Eur J Psychol Educ.

[45] Widder-Prewett R, Draime JA, Cameron G, Anderson D, Pinkerton M, Chen AMH (2017). Impact of student vs faculty facilitators on motivational interviewing student outcomes. Am J Pharm Educ.

