# Supplementary material for: Evaluating the effects of behavior change training on the knowledge, confidence and skills of sport and exercise science students
Source: BMC Sports Sci Med Rehabil. 2020 Oct 6;12:62. doi: 10.1186/s13102-020-00209-5 (PMC7539374; doi:10.1186/s13102-020-00209-5)
Supplement: Supplementary file 1 — Additional file 1. [file 13102_2020_209_MOESM1_ESM.docx]

**Table 1. Assessment methods to evaluate the effectiveness of the training program**

| **Kirkpatrick model level** | **Research aim of the level** | **Method of measurement within the program** | **Data collection method and time-point** | **Outcome measure used** |
| --- | --- | --- | --- | --- |
| 1. **Reaction**   How participants reacted to the program. | Were students satisfied with the program? | 1. Students self-reported satisfaction with the program. | Paper based questionnaire, post-program completion. | A study specific satisfaction questionnaire  *Validity*: Questions followed the suggestions of the Kirkpatrick model and previous research.  *Reliability:* Not applicable  *Sample question:* “I found the in-class activities (e.g., worksheets, group activities, case studies, videos) useful for developing my knowledge of how to use the SDT-based communication strategies.”; 1 “*strongly disagree*” to 5 “*strongly agree*”. |
| 1. **Learning**   The extent to which students change their attitudes, improve their knowledge, and/or increase their skills as a result of the program. | Did students’ knowledge, confidence and skills in components of behavior change improve following the program? | 1. Students’ self-reported confidence in behavior change components. | Paper based questionnaire, pre and post-program. | An intervention-specific self-reported confidence questionnaire  *Validity:* Questions followed the suggestions of the Kirkpatrick model.  *Reliability:* Not applicable.  *Sample question: “*How would you describe your confidence to select appropriate behavior change techniques to target psychological, social or environmental constructs?”; 1 “*not at all confident*” to 7 “*very confident*”. |
|  |  |  |  |  |
|  |  | 1. Students’ knowledge of SDT-based communication strategies. | Paper based questionnaire, pre and post-program. | An intervention-specific narrative questionnaire modelled on the Problems in Schools Questionnaire (Reeve, Bolt, & Cai, 1999)  *Validity:* Construct validity tested previously (Reeve et al., 1999).  *Reliability*: Tested for interrater agreement in this study using percentage agreement. The Problems in School Questionnaire has previously shown to have excellent interrater reliability (Reeve et al., 1999). |
|  |  | 1. Students’ knowledge of behavior change techniques (BCTs). | Paper based questionnaire, pre and post-program. | A sample intervention description extracted and adapted from the BCT taxonomy online training course.  *Validity*: This method of BCT training has been used successfully in previous studies (Wood et al., 2014).  *Reliability*: Tested for interrater agreement in this study using percentage agreement. |
|  |  | 1. Students’ skills in applying SDT-based communication strategies during an a hypothetical physical activity counselling session. | Audio-recorded interaction with a mock client assessed by an independent rater. | **The 6-item Health Care Climate Questionnaire (Williams et al., 1996)**  *Validity:* Construct validity has been tested in previous studies (Williams et al. 1996).  *Reliability*: This measure has been shown to have excellent interrater reliability (Murray et al., 2015). Reliability within this study was tested using intraclass correlation coefficients between the two blinded raters.  *Sample question: “*The practitioner listened to how the client would like to do things regarding being physically active or changing their behavior”; 1” *strongly disagree*” to 7 “*strongly agree*”.  **Intervention-specific measure of needs supportiveness adapted from Communication Evaluation in Rehabilitation Tool (CERT) (Murray et al., 2019)**  *Validity:* The original CERT scale has shown to have content validity (Murray et al., 2019).  *Reliability*: The original CERT scale has also been shown to have excellent interrater reliability. Reliability within this study was tested using intraclass correlation coefficients between the two blinded raters.  *Sample question: “*How well did the practitioner provide meaningful opportunities for client input into the conversation”; 1”not at all well” to 7 “very well’. |
|  |  |  | Student were asked to self-reflect on the interaction and their use of the SDT-based communication strategies during the hypothetical physical activity counselling session. | **Students’ reflections on using SDT-based communication strategies.**  *Validity:* Not applicable  *Reliability*: Not applicable |

**Table 2. Self-determination theory based-communication strategies ^a^**

| **Strategy** | **Description of strategy** |
| --- | --- |
| 1. Open ended questions | The practitioner did not use closed-ended questions at the beginning of a discussion. Instead the practitioner used open ended questions at the beginning of a discussion and where necessary closed-ended questions during follow-up questioning to obtain specific information. |
| 1. Staying silent | The practitioner allowed the client to complete sentences and finish speaking before following up with further questions. |
| 1. Summaries | After listening to the client, the practitioner summarised his / her perception of what the client had said. When done well the practitioner provided the client with the opportunity to confirm or clarify. |
| 1. Reflection ^b^ | The practitioner attempted to show the client that he / she understood the client’s perspective by reflecting back to the client what they said, either by simply repeating or slightly rephrasing what they had said or by making a guess as to what might come next. |
| 1. Asking permission | The practitioner asked the client if they were ready to consider advice regarding physical activity and / or the practitioner asked the client’s permission to give some information or advice. |
| 1. Providing a meaningful rationale | The practitioner explained to the client the rationale behind his / her advice. |
| 1. Opportunities for client input/choice | The practitioner asked the client for their opinion / input about their behavior change. In doing so, the client was given an opportunity to contribute to the conversation in a meaningful way. |
| 1. Autonomy supportive language | The practitioner attempted to motivate the client by supporting their autonomy in the situation e.g., by using suggestive and flexible language rather than using coercion or guilt inducing phrases. |
| 1. Goal-setting | Goals that are specific, measurable, achievable, and time-based were discussed by practitioner and client. These may not be named as “goals” in the conversation but are actions that the person is intending to carry out, and meet some or all of the SMART criteria. |
| 1. Barrier identification | The practitioner and client discussed a likely barrier (or barriers) to following advice or changing behavior. |
| 1. Solution identification | The practitioner and client brainstormed about how they might overcome this barrier. |

^a^ There were seven strategies listed in the Communication in Rehabilitation Evaluation Tool that were excluded from this study. These were: Use single questions; Catering for different learning preferences; Closing the loop; Provision of a rehabilitation diary, Contact details; Follow-up.

^b^ In this study, the strategy of empathy listed in the CERT was renamed and adapted to reflection.

**Table 3. Changes in students’ confidence** **following completion of the program**

| **Component** | **Median**  **pre-training**  **(IQR; min-max)** | **Median**  **post-training**  **(IQR; min-max)** | **Z score^a^** | | **p-value^c^** |
| --- | --- | --- | --- | --- | --- |
| **Confidence total [1 “*not confident at all*” to 7 “*very confident*”]^b^** | **3.6 (1.5; 1.7-5.7)** | **4.9 (1.3; 3.7-6.2)** | | **2.5** | **0.013** |
| 1. How would you describe your confidence to recognise and apply an appropriate theory to guide an intervention? | 2.0 (2.5; 1.0-5.0) | 4.0 (2.0; 3.0-6.0) | | 3.0 | 0.003* |
| 1. How would you describe your confidence to select appropriate behavior change techniques to target psychological, social or environmental constructs? | 2.0 (2.0; 1.0-5.0) | 6.0 (2.0; 3.0-6.0) | | 2.9 | 0.004* |
| 1. How would you describe your confidence to use active listening with clients? | 4.0 (2.0; 1.0-7.0) | 6.0 (1.5; 3.0-7.0) | | 1.9 | 0.06 |
| 1. How would you describe your confidence to assess how well an intervention is delivered? | 4.0 (2.5; 2.0-6.0) | 5.0 (2.0; 2.0-7.0) | | 1.7 | 0.09 |
|  |  |  | |  |  |

^a^ Difference between pre and post-program results;

^b^ Pre and post-confidence results relate to 14 of 15 students, as a result of missing data;

^C^ Nominal data therefore McNemar’s test calculated; *: p<0.007 is significant following Bonferroni correction;
